# Anti-Inflammatory Effects of Dietary Plant Stanol Supplementation Are Largely Dependent on the Intake of Cholesterol in a Mouse Model of Metabolic Inflammation

**DOI:** 10.3390/biomedicines9050518

**Published:** 2021-05-06

**Authors:** Inês Magro dos Reis, Tom Houben, Marion J. J. Gijbels, Dieter Lütjohann, Jogchum Plat, Ronit Shiri-Sverdlov

**Affiliations:** 1 Department of Molecular Genetics, School of Nutrition and Translational Research in Metabolism (NUTRIM), Maastricht University, 6229 ER Maastricht, The Netherlands; ines.reis@maastrichtuniversity.nl (I.M.d.R.); tom.houben@maastrichtuniversity.nl (T.H.); 2 Department of Pathology, Cardiovascular Research Institute Maastricht (CARIM,), School for Oncology and Developmental Biology (GROW), Maastricht University, 6229 ER Maastricht, The Netherlands; m.gijbels@maastrichtuniversity.nl; 3 Department of Medical Biochemistry, Experimental Vascular Biology, Amsterdam UMC, University of Amsterdam, 1105 AZ Amsterdam, The Netherlands; 4 Institute of Clinical Chemistry and Clinical Pharmacology, Venusberg-Campus 1, University Hospital Bonn, D-53127 Bonn, Germany; dieter.luetjohann@ukbonn.de; 5 Department of Nutrition and Movement Sciences, School for Nutrition, Toxicology and Metabolism (NUTRIM), Maastricht University, 6229 ER Maastricht, The Netherlands; j.plat@maastrichtuniversity.nl

**Keywords:** plant stanols, cholesterol, diet, hepatic inflammation

## Abstract

The prevalence of metabolic disorders characterized by chronic inflammation has been on a sharp rise for decades. As such, tools that address metabolic and inflammatory dysregulation are of great importance. Plant stanols are well-known for reducing intestinal cholesterol absorption and may also have direct anti-inflammatory effects. In this study, our aim was to investigate to what extent the benefits of dietary plant stanol supplementation depend on dietary cholesterol intake in an experimental mouse model for cholesterol-induced metabolic inflammation. Here, we used *Ldlr^−/−^* mice transplanted with *Npc1^nih^*-derived bone marrow, featuring feature bone marrow-derived immune cells characterized by chronic inflammation induced by lysosomal lipid accumulation. *Npc1^nih^*- and *Npc1^wt^*-transplanted mice were placed on either a high fat, high cholesterol (HFC) or on a chow diet low in cholesterol, with or without 2% plant stanols supplementation. At the end of the study, the metabolic and inflammatory status of the mice was analyzed. Plant stanol supplementation to the HFC diet reduced liver cholesterol levels and improved lipid metabolism and liver inflammation, particularly in *Npc1^nih^*-tp mice. In contrast, plant stanol supplementation to the chow diet did not significantly improve the aforementioned parameters, though similar reductive trends to those in the HFC diet setting were observed regarding liver cholesterol accumulation and liver inflammatory markers. The effects of dietary plant stanol supplementation on dietary cholesterol-induced inflammation are largely dependent on dietary cholesterol intake. Future research should verify whether other models of metabolic inflammation exhibit similar stanol-related effects on inflammation.

## 1. Introduction

Despite efforts to raise awareness on the importance of a healthy lifestyle, the prevalence of obesity accompanied by metabolic syndrome has been on a sharp rise for decades [1]. Individuals who meet at least three of the following criteria are diagnosed with metabolic syndrome: increased hip-to-waist ratio, hypertriglyceridemia, hyperglycemia, low high-density lipoprotein plasma levels, and hypertension. Individuals who suffer from metabolic syndrome are at high risk of developing severe non-communicable diseases such as type II diabetes mellitus, atherosclerosis, liver and cardiovascular diseases, cancer, and even dementia and depression. Not only are the aforementioned diseases associated with reduced quality of life, they also account for nearly 70% of all premature deaths worldwide [2]. Overall, it is clear that a concerted effort is required to increase the awareness of the strengths of lifestyle-related prevention strategies as well as to develop efficient strategies to target metabolic syndrome and its associated disease burden.

Plant stanols are plant-derived molecules that result from the saturation of plant sterols, which are analogous to cholesterol in structure. Consumption of two to three grams per day of plant stanols is well-known to interfere with intestinal cholesterol absorption, thereby lowering plasma cholesterol levels and as such reducing inflammatory cascades [3]. In addition, studies suggest that plant stanols also have immunomodulatory and anti-inflammatory effects [4,5], and it has been suggested that these effects might occur independently from their ability to reduce cholesterol absorption [4]. However, this assumption of independency has yet to be validated in in vivo models for metabolic diseases. Previously, our group showed that dietary plant stanol supplementation reduces hepatic cholesterol levels, ameliorates liver inflammation, and shifts blood immune cells towards a less pro-inflammatory profile in a murine model of Niemann–Pick type C1 (NPC1) disease [5]. NPC1 disease is caused by deleterious mutations in the NPC1 gene that lead to the production of a defective NPC1 protein, a lysosomal cholesterol efflux transporter [6]. Following impaired NPC1 protein function, lysosomal cholesterol and sphingolipid accumulation occurs in all tissues, culminating in increased oxidative stress and severe inflammation. Importantly, in the aforementioned study [5], mice were fed a diet with much lower cholesterol content than in an experimental high fat, high cholesterol (HFC) diet (0.37 µg vs. 1.62 µg cholesterol/mg chow on average). As such, this study puts forward the hypothesis that plant stanols have anti-inflammatory effects independent of reduced cholesterol intestinal absorption, although such effects have not been evaluated yet side-by-side on a background of either cholesterol-rich or cholesterol-poor diets.

Therefore, in the current study, our aim was to investigate to what extent the effects of dietary plant stanol supplementation on hepatic and systemic cholesterol metabolism and inflammation depend on dietary cholesterol intake. To this end, we analyzed the effects of two-percent dietary plant stanol supplementation on either an HFC or a chow diet in a model for cholesterol-induced inflammation. Here, we used low-density lipoprotein receptor knockout (*Ldlr^−/−^*) mice, which, when fed an HFC diet, display hypercholesterolemia and increased hepatic cholesterol accumulation and inflammation, thus mimicking the human situation of metabolic diseases such as atherosclerosis and non-alcoholic steatohepatitis [7,8]. In order to induce hepatic lipid accumulation and inflammation in the absence of a HFC diet, we transplanted *Ldlr^−/−^* mice with *Npc1^nih^* bone marrow (*Npc1^nih^*-tp mice) [9]. These *Npc1^nih^*-tp mice display increased levels of hepatic inflammation compared to their *Npc1^wt^*-tp counterparts, even in the absence of a HFC diet, thus serving as a model to investigate the influence of diet on the anti-inflammatory properties of plant stanols.

## 2. Materials and Methods

### 2.1. Mice, Bone Marrow Transplant, and Diet

Throughout the study, mice were housed under standard conditions and had unlimited access to food and water, unless explicitly mentioned otherwise. For one week prior to and up to four weeks after irradiation, *Ldlr^−/−^* mice were housed in filter-top cages and received antibiotics diluted in drinking water to prevent infections following immunosuppression (Neomycin, 100 mg/L, Gibco, Breda, the Netherlands; 6 × 10^4^ U/L polymycin B sulphate). Six-week-old bone marrow from *Npc1^nih^* and *Npc1^wt^* mice donors were derived from heterozygous founders of a C57BL/6 genetic background. The genotype of *Npc1^nih^* and *Npc1^wt^* mice was determined as previously described [10]. On the day of bone marrow transplant, *Npc1^nih^* and *Npc1^wt^* littermates were sacrificed via CO_2_ inhalation and their bone marrows were isolated. On the day before and on the day of bone marrow transplant, *Ldlr^−/−^* mice were subjected to six Gray of γ-radiation, thus having received 12 Gray of γ-radiation before receiving 1 × 10^7^ bone marrow cells collected from *Npc1^wt^* or *Npc1^nih^* mice via intravenous injection.

**Figure 1:**
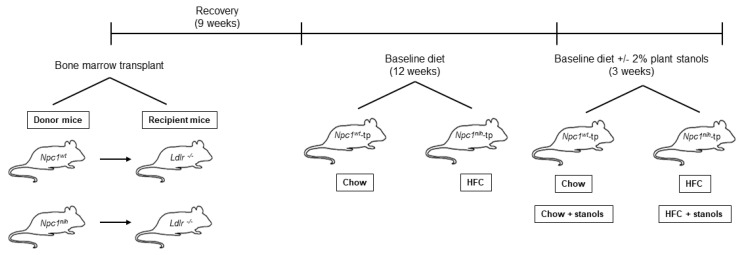
Overview of study setup and timeline. *Ldlr^−/−^* mice received a bone marrow transplant from *Npc1^nih^* or *Npc1^wt^* mice. After recovery, *Npc1^wt^*-tp and *Npc1^nih^*-tp mice received a cholesterol-poor chow or HFC diet for 12 weeks. On the final three weeks of the study period, their diet was supplemented with 2% plant stanols.

After nine weeks of recovery, transplanted mice were placed on a chow or HFC diet for twelve weeks, after which they were assigned to two different experimental groups and received the corresponding plant stanol experimental diets (Table S1) for three weeks, for a total of eight experimental groups (*n* = 6–8 mice per group): (1) *Npc1^wt^*-tp and *Npc1^nih^*-tp mice on a regular chow diet; (2) *Npc1^wt^*-tp and *Npc1^nih^*-tp mice on a two percent plant stanol-enriched chow diet; (3) *Npc1^wt^*-tp and *Npc1^nih^*-tp mice on a HFC diet; (4) *Npc1^wt^*-tp and *Npc1^nih^*-tp mice on a two percent plant stanol-enriched HFC diet. For a schematic overview of the study setup and timeline, please refer to Figure 1. All experiments were performed according to Dutch laws and approved by the Animal Experiment Committee of Maastricht University (study DEC 2013-002, approved on 19 March 2013).

### 2.2. Bone Marrow Transplant Efficiency

In order to assess bone marrow transplant efficiency, we determined chimerism in *Ldlr^−/−^* mice transplanted with bone marrow from *Ldlr^wt^* mice. Genomic DNA was isolated using the PureLink Genomic DNA, according to the manufacturer’s instructions (K182002; ThermoFisher Scientific, Waltham, MA, USA). A standard curve was built by producing solutions with different ratios of *Ldlr*^−/−^ and *Ldlr^wt^* bone marrow DNA. To assess chimerism of mice in this study, we analyzed the amount of *Ldlr^−/−^* DNA in 70 µL peripheral blood samples. To standardize the total amount of DNA among different samples, the *p50* gene expression was quantified. Samples were assayed in duplicate on a 7900HT real-time PCR system by using 25 ng DNA and a SensiMixTM Sybr & Fluorescein kit (QT615-05, Bioline, Memphis, Tennessee, U.S.A.), according to the manufacturer’s instructions. *Ldlr^−/−^*-specific primers are forward 5’-GCTGCAACTCATCCATATGCA-3’ and reverse 5’-GGAGTTGTTGACCTCGACTCTAGAG-3’. Forward and reverse *p50*-specific primers are 5’-ACCTGGGAATACTTCATGTGACTAA-3’ and 5’-ACACCAGAAGTCCAGGATTATCAG3’, respectively. A standard curve was generated by plotting the mean threshold cycle (Ct) ΔCt (Ct *p50*—Ct *Ldlr*^−/−^) against the logarithm of the percentage *Ldlr*^−/−^ and calculation of a regression line. Bone marrow transplant efficiency was determined as the percentage of *Ldlr*^−/−^ DNA in the mice blood samples (representing the remaining recipient bone marrow), calculated by applying the mean ΔCt of the sample to the previously generated standard curve. On average, bone marrow transplantation was nearly 94% efficient (Table S2), indicating that the procedure was successful.

### 2.3. Lipid and Gene Expression Analyses

Upon sacrifice, all tissues were isolated and snap-frozen in liquid nitrogen and stored at −80 °C or fixed in 4% formaldehyde/PBS. The collection of blood and tissue specimens, biochemical determination of lipids in plasma, RNA isolation, cDNA synthesis, and qPCR were determined as described previously [11,12,13]. Hepatic sterol content was determined by gas-liquid chromatography–mass spectroscopy, as described elsewhere [14].

### 2.4. Immunohistochemistry

Frozen liver sections (7 µm) were fixed in acetone and blocked for endogenous peroxidase by incubation with 0.25% of 0.03% H_2_O_2_ for 5 min. Primary antibodies used were against hepatic macrophages (1:100 rat anti-mouse CD68, clone FA11), infiltrated macrophages and neutrophils (1:500 rat anti-mouse Mac-1 (M1/70)), and infiltrated T-cells (1:20, rat anti-mouse CD3). 3-Amino-9-ethylcarbazole was applied as color substrate and hematoxylin for nuclear counterstain. Sections were enclosed with Faramount aqueous mounting medium.

Pictures were taken with a Nikon digital camera DMX1200 and ACT-1 v2.63 software (Nikon Instruments Europe, Amstelveen, The Netherlands). Infiltrated macrophages and neutrophil cells (Mac-1+) and infiltrated T-cells (CD3+) were counted by two blinded researchers in six microscopical views (original magnification, 200×) and were indicated as number of cells per square millimeter (cells/mm^2^). Hepatic macrophages (CD68) were counted in six microscopical views (original magnification, 200×) and indicated as the percentage of CD68 positive area (Adobe Photoshop CS2 v.9.0., San Jose, CA, USA).

### 2.5. Plasma FACS Analyses

Tail vein blood was collected from mice 12 and 15 days after the beginning of the plant stanol-enriched diet. FACS procedures were performed as previously described [5].

### 2.6. Statistical Analysis

Data were statistically analyzed by performing two-way ANOVA and Tukey’s post hoc test using GraphPad Prism software (version 6 for Windows, GraphPad Software Inc, San Diego, CA, USA). Data were expressed as the group mean and standard error of the mean. Statistical significance is indicated on the following data comparisons: *Npc1^wt^*-tp or *Npc1^nih^*-tp mice on a chow or HFC diet vs. *Npc1^wt^*-tp or *Npc1^nih^*-tp mice on a plant stanol-enriched chow or HFC diet (* *p* ≤ 0.05; ** *p* < 0.01; *** *p* < 0.001; **** *p* < 0.0001). *Npc1^nih^*-tp mice on a chow or HFC diet vs. *Npc1^wt^*-tp mice on a chow or HFC diet (# *p* ≤ 0.05; ## *p* < 0.01; ### *p* < 0.001; #### *p* < 0.0001).

## 3. Results

### 3.1. Plant Stanol Supplementation Improves Lipid Metabolism in Npc1^nih^-tp Mice on a HFC and to a Lesser Extent in Mice on a Chow Diet

**Figure 2:**
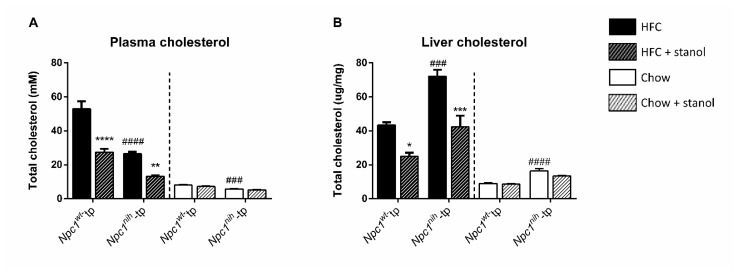
Plasma and liver lipid levels: HFC and chow diets. (**A**) Total plasma cholesterol levels and (**B**) total liver cholesterol levels. *n* = 6–8 mice per group. All error bars represent standard error of the mean. Statistical significance is indicated as follows: *Npc1^nih^*-tp mice on an HFC or chow diet vs. *Npc1^wt^*-tp mice on an HFC or chow diet (### *p* < 0.001; #### *p* < 0.0001); *Npc1^wt^*-tp or *Npc1^nih^*-tp on a HFC or chow diet vs. *Npc1^wt^*-tp or *Npc1^nih^*-tp mice on a plant stanol-enriched HFC or chow diet (* *p* ≤ 0.05; ** *p* < 0.01; *** *p* < 0.001; **** *p* < 0.0001).

To determine to what extent the effects of plant stanols on lipid metabolism are dependent on diet, we analyzed plasma and liver cholesterol levels of *Npc1^wt^*-tp mice and of *Npc1^nih^*-tp mice placed on an HFC or chow diet for 15 weeks. At baseline, *Npc1^nih^*-tp mice displayed lower plasma total cholesterol but higher hepatic cholesterol concentrations as compared to the *Npc1^wt^*-tp mice, both following HFC and chow diets (Figure 2).

As expected, adding plant stanols to the HFC diet effectively reduced plasma and hepatic cholesterol levels in both experimental groups (Figure 2), whereas plant stanol supplementation of the chow diet had no significant effect on plasma and liver cholesterol levels in either experimental group (Figure 2).

**Figure 3:**
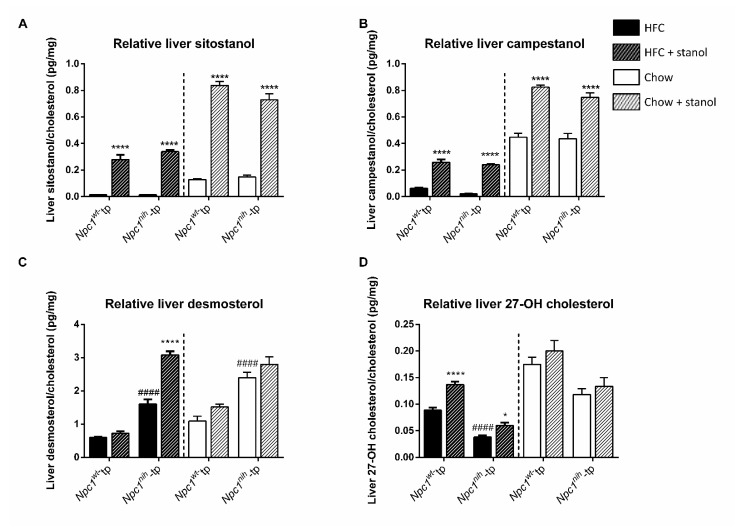
Hepatic sterol lipid metabolism: HFC and chow diets. Liver levels of (**A**) sitostanol, (**B**) campestanol, (**C**) desmosterol, and (**D**) 27-OH cholesterol relative to total hepatic cholesterol levels. *n* = 6–8 mice per group. All error bars represent standard error of the mean. Statistical significance is indicated as follows: *Npc1^nih^*-tp mice on an HFC or chow diet vs. *Npc1^wt^*-tp mice on a HFC or chow diet (#### *p* < 0.0001); *Npc1^wt^*-tp or *Npc1^nih^*-tp on a HFC or chow diet vs. *Npc1^wt^*-tp or *Npc1^nih^*-tp mice on a plant stanol-enriched HFC or chow diet (* *p* ≤ 0.05; **** *p* < 0.0001).

To have a deeper insight into hepatic cholesterol metabolism following plant stanol supplementation, we assessed liver levels of the following sterols in relation to liver cholesterol levels: sitostanol and campestanol, which were included in the plant stanol-supplemented diet and can be seen as compliance markers; desmosterol, a cholesterol biosynthesis precursor; and 27-OH cholesterol, a bile acid precursor. After three weeks of plant stanol supplementation to HFC or chow diet, relative hepatic sitostanol and campestanol levels increased in both chimeric groups, as expected (Figure 3A,B). Following an HFC diet, increased relative desmosterol levels and reduced 27-OH cholesterol were observed in *Npc1^nih^*-tp mice compared to *Npc1^wt^*-tp mice (Figure 3C,D). Regarding the chow diet setting, relative liver desmosterol levels were also increased in *Npc1^nih^*-tp mice compared to *Npc1^wt^*-tp mice, whereas relative 27-OH cholesterol levels tended to be reduced in *Npc1^nih^*-tp mice compared to *Npc1^wt^*-tp mice (Figure 3C,D).

While supplementing the HFC diet with plant stanols had no effect on relative desmosterol levels in the livers of *Npc1^wt^*-tp mice, *Npc1^nih^*-tp mice displayed increased relative desmosterol levels following increased plant stanol intake (Figure 3C). The aforementioned results suggest that, following a HFC diet, plant stanol supplementation increases liver cholesterol synthesis in *Npc1^nih^*-tp mice, but not in *Npc1^wt^*-tp mice. Furthermore, plant stanol supplementation to an HFC diet increased relative liver levels of 27-OH cholesterol in both chimeric groups, suggesting increased metabolism of cholesterol to oxidized sterols acids following increased plant stanol intake (Figure 3D). In contrast to the HFC setting, plant supplementation to a chow diet had no effects on hepatic levels of desmosterol and 27-OH cholesterol in either chimeric group (Figure 3D).

**Figure 4:**
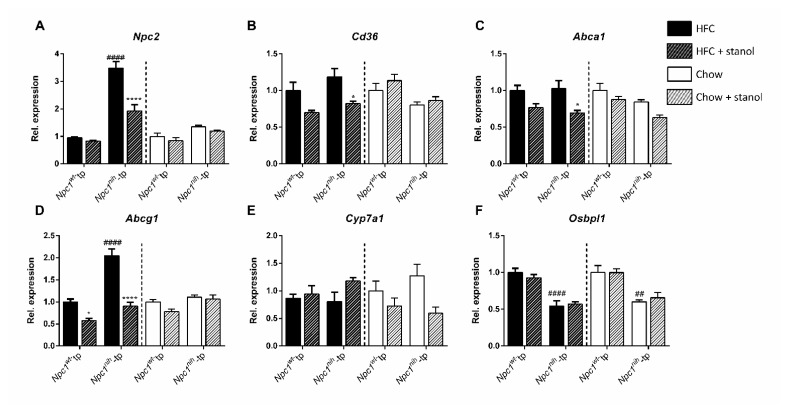
Hepatic lipid metabolism-related gene expression: HFC and chow diets. Hepatic gene expression levels of (**A**) *Npc2*, (**B**) *Cd36*, (**C**) *Abca1*, (**D**) *Abcg1*, (**E**) *Cyp7a1*, and (**F**) *Osbpl1*. *n* = 6–8 mice per group. All error bars represent standard error of the mean. Statistical significance is indicated as follows: *Npc1^wt^*-tp mice on a HFC or chow diet vs. *Npc1^wt^*-tp mice on a HFC or chow diet (## *p* < 0.01; #### *p* < 0.0001); *Npc1^nih^*-tp or *Npc1^nih^*-tp on a HFC or chow diet vs. *Npc1^wt^*-tp or *Npc1^nih^*-tp mice on a plant stanol-enriched HFC or chow diet (* *p* ≤ 0.05; **** *p* < 0.0001).

To further assess the effects of plant stanol supplementation in liver lipid metabolism, we analyzed the liver expression of the following genes: *Npc2*, which encodes for a protein that transfers luminal cholesterol in late endosomes/lysosomes to NPC1; *Cd36*, a receptor for modified LDL, a pro-inflammatory lipoprotein; *Abca1* and *Abcg1*, which mediate HDL synthesis and excess cholesterol efflux; *Cyp7a1*, a bile acid synthesis mediator; and *Osbpl1*, a cytosolic protein that binds oxygenated forms of cholesterol (Figure 4). Following an HFC diet, *Npc1^nih^*-tp mice displayed higher *Npc2* and *Abcg1* expression but lower *Osbpl1* expression as compared to the *Npc1^wt^*-tp mice (Figure 4A,D,F). Regarding the chow diet setting, while expression of most analyzed genes was comparable between the experimental groups, hepatic *Npc2* expression tended to be increased in *Npc1^nih^*-tp mice, whereas *Osbpl1* expression was effectively reduced in *Npc1^nih^*-tp mice compared to *Npc1^wt^*-tp mice (Figure 4F).

Adding plant stanols to the HFC diet reduced *Npc2*, *Cd36*, *Abca1*, and *Abcg1* hepatic expression in *Npc1^nih^*-tp mice, suggesting reduced lysosomal lipid accumulation, modified cholesterol uptake, and excess cholesterol efflux (Figure 4A–D). Furthermore, increased plant stanol consumption also reduced *Abcg1* expression and tended to reduce hepatic *Cd36* expression in the livers of *Npc1^wt^*-tp mice on a HFC diet, although the latter effect did not reach statistical significance (Figure 4B,D). Following plant stanol supplementation to the chow diet, no significant differences were observed in any of the analyzed hepatic genes’ expression. Altogether, the results described above indicate that dietary plant stanol supplementation only has minor beneficial effects on lipid metabolism in the absence of a HFC diet in *Npc1^nih^*-tp mice.

### 3.2. Dietary Plant Stanol Supplementation Has Little Impact on Liver Inflammation in the Absence of a HFC Diet

**Figure 5:**
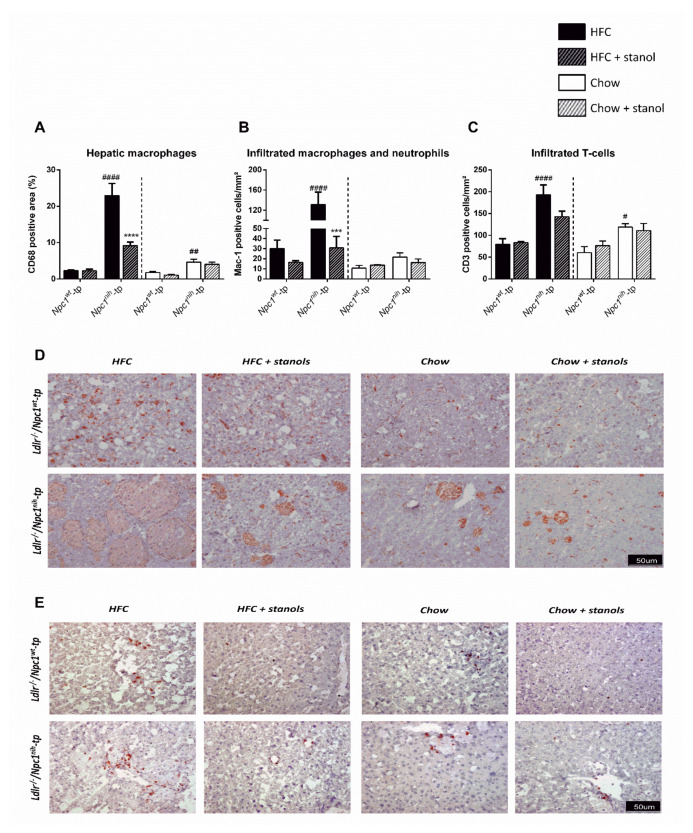
Hepatic inflammation-related immunohistochemistry: HFC and chow diets. (**A**–**C**) Quantification of hepatic macrophages (CD68), infiltrated hepatic macrophages and neutrophils (Mac-1), and infiltrated hepatic T-cells (CD3). (**D**,**E**) Representative pictures of liver sections stained for hepatic macrophages and infiltrated macrophages. All error bars represent standard error of the mean. Statistical significance is indicated as follows: *Npc1^nih^*-tp mice on a HFC or chow diet vs. *Npc1^wt^*-tp mice on a HFC or chow diet (# *p* ≤ 0.05; ## *p* < 0.01; #### *p* < 0.0001); *Npc1^wt^*-tp or *Npc1^nih^*-tp on a HFC or chow diet vs. *Npc1^wt^*-tp or *Npc1^nih^*-tp mice on a plant stanol-enriched HFC or chow diet (*** *p* < 0.001; **** *p* < 0.0001).

To investigate whether plant stanol supplementation reduces liver inflammation in the absence of a beneficial plant stanol-induced metabolic effect, we measured hepatic macrophages (CD68^+^) and infiltrated macrophages and neutrophils (Mac-1^+^), as well as infiltrated T-cells (CD3^+^) via immunohistochemistry (Figure 5). Following an HFC diet, *Npc1^nih^*-tp mice displayed higher numbers of hepatic macrophages, infiltrated macrophages, neutrophils, and T-cells as compared to the *Npc1^wt^*-tp mice (Figure 5). Regarding the chow diet setting, while no differences were observed regarding infiltrated macrophages and neutrophils between experimental groups, *Npc1^nih^*-tp mice displayed increased presence of hepatic macrophages and infiltrated T-cells compared to *Npc1^wt^*-tp mice, suggesting increased liver inflammation following *Npc1* mutation in bone marrow-derived immune cells (Figure 5).

As expected, while plant stanol supplementation to HFC diet had no effect on hepatic levels of immune cells in *Npc1^wt^*-tp mice, dietary plant stanol supplementation reduced the amount of hepatic macrophages and infiltrated macrophages and neutrophils in *Npc1^nih^*-tp mice (Figure 5). In addition, *Npc1^nih^*-tp mice on a plant stanol-supplemented HFC diet showed a tendency for reduced infiltrated T-cells, although this effect did not reach statistical significance (Figure 5C). Supplementing the mice’s chow diet with plant stanols had no effect on the hepatic levels of any of the immune cells in either experimental group (Figure 5).

**Figure 6:**
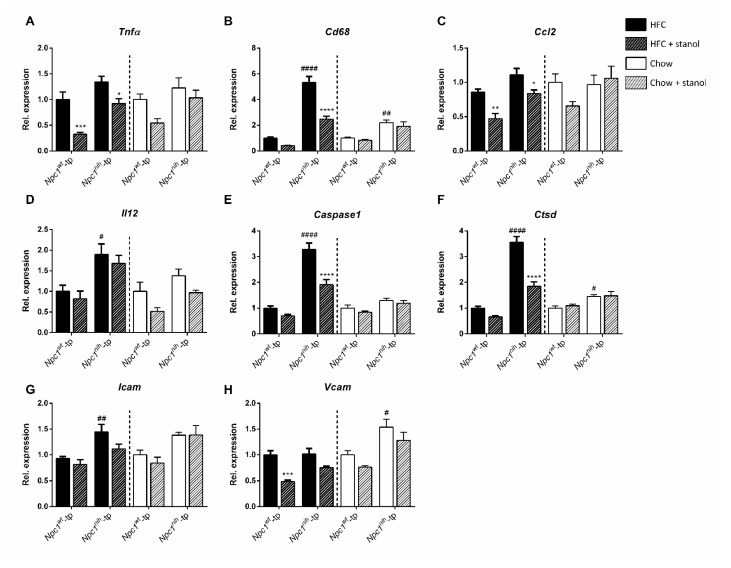
Hepatic inflammation-related gene expression: HFC and chow diets. Hepatic gene expression of inflammatory markers (**A**) *Tnf-α*, (**B**) *Cd68*, (**C**) *Ccl2*, (**D**) *Il12*, (**E**) *Caspase1*, (**F**) *Ctsd*, (**G**) *Icam* and (**H**) *Vcam*. *n* = 6–8 mice per group for gene expression analyses. All error bars represent standard error of the mean. Statistical significance is indicated as follows: *Npc1^nih^*-tp mice on a HFC or chow diet vs. *Npc1^wt^*-tp mice on a HFC or chow diet (# *p* ≤ 0.05; ## *p* < 0.01; #### *p* < 0.0001); *Npc1^wt^*-tp or *Npc1^nih^*-tp on a HFC or chow diet vs. *Npc1^wt^*-tp or *Npc1^nih^*-tp mice on a plant stanol-enriched HFC or chow diet (* *p* ≤ 0.05; ** *p* < 0.01; *** *p* < 0.001; **** *p* < 0.0001).

To profile the effects of plant stanols on hepatic inflammation in more detail, we analyzed the expression of the following genes: *Tnfα*, *Cd68*, *Ccl2*, *Il12*, *Caspase1*, *Ctsd*, *Icam*, and *Vcam* (Figure 6). Following an HFC diet, *Npc1^nih^*-tp mice displayed increased expression of *Cd68*, *Il12*, *Caspase1*, *Ctsd*, and *Icam* compared to *Npc1^nih^*-tp mice (Figure 6B,D–G). In addition, *Ccl2* expression tended to be increased in *Npc1^nih^*-tp mice compared to Npc1nih-tp mice, although this effect did not reach statistical significance (Figure 6C). Regarding the chow setting, *Npc1^nih^*-tp mice on a chow diet displayed increased hepatic expression of *Cd68*, *Ctsd*, and *Vcam* compared to *Npc1^wt^*-tp mice (Figure 6B,F,H). In line with our immunohistochemical observations, these results indicate increased hepatic inflammation in *Npc1^nih^*-tp mice compared to *Npc1^nih^*-tp mice, particularly in the HFC diet setting.

Following plant stanol supplementation to the HFC diet, expression of *Tnfα* and *Ccl2* was reduced in both experimental groups (Figure 6A,C). Furthermore, *Npc1^nih^*-tp mice displayed reduced expression of *Cd68*, *Caspase1*, and *Ctsd*, as well as a tendency for reduced *Vcam* expression, following a plant stanol-enriched HFC diet (Figure 6B,E,F,H). Finally, we observed reduced expression of *Vcam* in the livers of *Npc1^wt^*-tp mice on a plant stanol-enriched HFC diet compared to *Npc1^wt^*-tp mice on a HFC diet (Figure 6H). Similarly to immunohistochemistry results, plant stanol supplementation to the chow diet had no impact on the expression of the analyzed genes in either genotype (Figure 6). Overall, the results described above indicate that, in line with lipid metabolism results, plant stanol supplementation had fewer effects on liver inflammation in *Npc1^nih^*-tp mice fed a chow diet compared to *Npc1^nih^*-tp mice fed a HFC diet.

**Figure 7:**
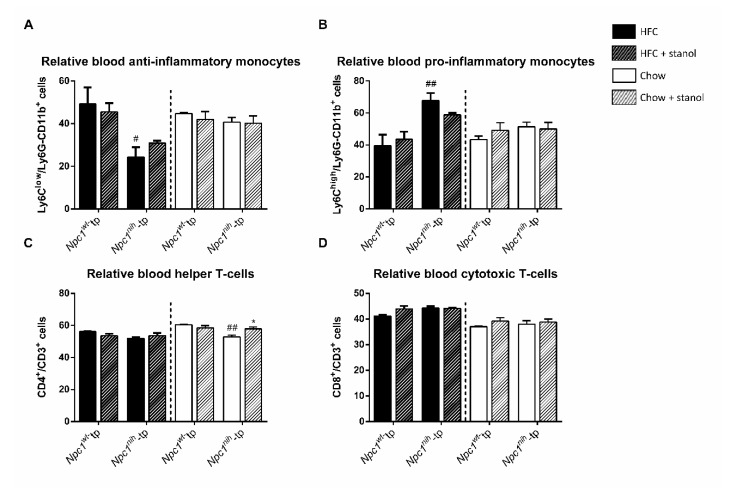
Phenotype and levels of blood monocytes and T-cells: HFC and chow diets. Relative levels of plasma (**A**) pro-inflammatory (LyC6+) and (**B**) anti-inflammatory (LyC6−) monocytes, as well as of (**C**) cytotoxic (CD8+) and (**D**) helper T-cells (CD4+) were measured by FACS analysis. *n* = 4 mice per group. All error bars represent standard error of the mean. Statistical significance is indicated as follows: *Npc1^nih^*-tp mice on a HFC or chow diet vs. *Npc1^wt^*-tp mice on a HFC or chow diet (# *p* ≤ 0.05; ## *p* < 0.01); *Npc1^wt^*-tp or *Npc1^nih^*-tp on a HFC or chow diet vs. *Npc1^wt^*-tp or *Npc1^nih^*-tp mice on a plant stanol-enriched HFC or chow diet (* *p* ≤ 0.05).

Finally, we analyzed the blood monocytes and T-cells three weeks after the start of plant stanol supplementation to further investigate the effects of plant stanol supplementation on systemic inflammation (Figure 7). In this study, we analyzed the relative amounts of blood LyC6^+^ cells (a marker for activated pro-inflammatory monocytes), as well as of CD4^+^ and CD8^+^ cells, which denote helper and cytotoxic T-cells, respectively. While no differences were observed on T-cell populations among genotypes following an HFC diet (Figure 7C,D), *Npc1^nih^*-tp mice displayed lower levels of anti-inflammatory LyC6^low^ monocytes and higher levels of pro-inflammatory LyC6^high^ monocytes than *Npc1^wt^*-tp mice, indicating increased systemic inflammation in the former genotype following an HFC diet (Figure 7A,B). Regarding the chow setting, although *Npc1^nih^*-tp mice displayed reduced relative levels of blood T-helper cells compared to *Npc1^wt^*-tp mice (Figure 7C), relative amounts of blood anti- and pro-inflammatory monocytes and cytotoxic T-cells were comparable between chimeric groups following a chow diet (Figure 7A,B,D).

Following plant stanol enrichment to the HFC diet, no significant effects were observed on any of the analyzed blood immune cell populations in either genotype (Figure 7). Of note, plant stanol supplementation to the HFC diet tended to increase levels of anti-inflammatory monocytes and to reduce levels of pro-inflammatory monocytes in *Npc1^nih^*-tp mice, although this effect did not reach statistical significance. After three weeks of plant stanol supplementation to the chow diet, we observed increased amounts of CD4^+^ T-cells in *Npc1^nih^*-tp mice, suggesting that plant stanols increased the relative amount of blood helper T-cells (Figure 7C). Overall, despite the observed increase in helper T-cell levels in *Npc1^nih^*-tp mice on a plant stanol-enriched chow diet, these results suggest that three weeks of plant stanol supplementation has little impact on blood immune cell populations, regardless of genotype or diet.

Altogether, the results described in this study indicate that beneficial effects of dietary plant stanol supplementation on lipid metabolism and inflammation are mostly dependent on excess intake of cholesterol in the analyzed model.

## 4. Discussion

Although glucose and lipid metabolism dysregulation are at the core of the metabolic syndrome, the ensuing inflammatory response underlies the development of metabolic syndrome-related diseases, such as atherosclerosis and NASH. As such, tools to prevent or reduce inflammation are paramount for metabolic syndrome patients. Plant stanols are well-known to reduce dietary cholesterol absorption and to consequently reduce plasma cholesterol levels and inflammation. However, reports also suggest that plant stanols may have direct anti-inflammatory effects of their own, increasing their appeal as affordable, easily accessible anti-inflammatory tools for individuals with inflammatory disorders who do not consume cholesterol in excess. In this study, only subtle trends towards improved metabolism and inflammation were observed following plant stanol supplementation to a cholesterol-poor diet. In contrast, plant stanol supplementation to the HFC diet clearly improved lipid metabolism and inflammation in *Npc1^nih^*-tp mice. Our results suggest that individuals who consume excessive amounts of cholesterol are likely to benefit the most from plant stanol supplementation.

For decades, plant stanols and their unsaturated analogues, plant sterols, have been known to interfere with intestinal cholesterol absorption and to reduce plasma cholesterol levels in animal models and humans alike. Consequently, a variety of plant stanol-supplemented foods have been developed, which are often recommended to (mildly) hypercholesterolemic patients as a tool to reduce their plasma cholesterol levels. While the mechanisms underlying the effects of plant stanols on intestinal cholesterol absorption are still a matter of debate, it is likely that this effect is accomplished by a variety of processes, including interference with chylomicron cholesterol incorporation and stimulation of intestinal cholesterol excretion, for instance via LXR activation [3]. In addition to stimulating cholesterol excretion in the intestines, it is possible that absorbed plant stanol molecules can likewise activate LXR signaling in the liver, thereby modulating hepatic lipid metabolism even in the absence of an HFC diet. Of note, experimental manipulations that were used in this experiment likely did not change intestinal NPC1L1 expression, excluding a role for this transporter in our study [3]. While our results strengthen the view that plant stanols’ effects on hepatic lipid metabolism are highly dependent on interference with intestinal cholesterol absorption, the aforementioned trends suggest that plant stanol molecules may have direct effects on hepatic lipid metabolism, for instance via LXR activation. Since only about 0.15% of ingested plant stanols is effectively absorbed into the circulation [15], it is possible that such direct effects would have translated into stronger modulation of hepatic lipid metabolism following supplementation with increased concentration of plant stanols to the chow diet during a longer time period. In addition to being well-known LXR activators, plant stanols also modulate sterol metabolism, which ultimately can influence hepatic inflammation. Relevantly, while in a previous study plant stanol supplementation reduced liver desmosterol levels in *Ldlr^−/−^* mice fed a HFC diet [16], here we observed a further increase in liver desmosterol levels of *Npc1^nih^*-tp mice following plant stanol supplementation to an HFC diet, and a similar trend was observed in the regular chow diet setting. While the physiological relevance of such an increase is unclear, considering the accompanying reduction in liver inflammation, it is possible that plant stanol supplementation stimulates *Npc1^nih^* macrophages to further accumulate desmosterol. Such a desmosterol accumulation could increase LXR activation and inhibit SREBP-pathways, thereby reducing the inflammatory profile of *Npc1^nih^* macrophages and possibly contributing to reduced inflammation following plant stanol supplementation [17,18].

In addition to interfering with intestinal cholesterol absorption and modulating lipid metabolism via diverse signaling pathways, there is further evidence indicating that plant stanols may have anti-inflammatory and immunomodulatory effects independent from their effects on cholesterol intestinal absorption. Previously, one in vitro study showed that sitostanol administration to bone marrow-derived macrophages reduced *TNFα* secretion in the absence of changes in *Lxr* expression [16], suggesting that plant stanols shift macrophages towards a less pro-inflammatory profile independently of LXR activation and of lipid metabolism status of other cell types, such as hepatocytes. Further studies showed that increased plant stanol consumption shifts T-cells towards a Th1 profile in asthma patients, a process likely mediated by TLR2 activation [19,20,21]. These findings suggest that plant stanols have immunomodulatory effects independent of effects on lipid metabolism. However, such effects have yet to be validated in in vivo models regarding metabolic inflammatory disorders, such as NASH, cardiovascular disease, and atherosclerosis. In the current study, apart from a modest increase in circulating helper T-cells in *Npc1^nih^*-tp mice, we observed only subtle reductions in hepatic inflammatory markers following plant stanol supplementation to a chow diet. It should be noted that, while *Npc1^nih^*-tp mice consistently displayed higher liver lipid accumulation and inflammation compared with *Npc1^wt^*-tp mice, the disease phenotype was much more subtle in *Npc1^nih^*-tp mice fed a chow diet than in the HFC diet setting. As such, it is possible that a stronger disease phenotype is needed in order to observe more pronounced effects of plant stanol supplementation. In addition, in a previous study [5], *Npc1^nih^* mice on a cholesterol poor diet showed strong improvements in systemic inflammation after five weeks of plant stanol supplementation, rather than three, as in the current study. Furthermore, while we observed improvements in the NPC1 disease phenotype following 2% plant stanol supplementation in this study [5], such effects were more pronounced in mice consuming a 6% plant stanol-enriched diet. Therefore, it is possible that administration of higher concentrations of plant stanols for a longer time period would also have elicited significant anti-inflammatory effects in the chow setting in the current study, as has also been proposed in previous discussions regarding contradicting observations of plant stanols’ anti-inflammatory effects in cardiovascular disease patients [22,23,24]. Overall, although we cannot fully exclude other direct, albeit subtle, anti-inflammatory effects, our findings indicate that plant stanols’ immunomodulatory effects are mainly visible in conditions of a high cholesterol intake. As such, while long-term plant stanol supplementation may benefit individuals with low-grade inflammation who do not consume excessive cholesterol, it is likely that plant stanols will bring the most benefits to patients with cholesterol-enriched, unhealthy diets.

## Data Availability

Data presented in this study can be requested from the corresponding author, Ronit-Shiri Sverdlov.

## Supplementary Materials

The following are available online at https://www.mdpi.com/article/10.3390/biomedicines9050518/s1, Table S1: Food composition; Table S2: Bone marrow transplant efficiency.
