# Supplementary material for: Anti-Inflammatory Effects of Dietary Plant Stanol Supplementation Are Largely Dependent on the Intake of Cholesterol in a Mouse Model of Metabolic Inflammation
Source: Biomedicines. 2021 May 6;9(5):518. doi: 10.3390/biomedicines9050518 (PMC8148209; doi:10.3390/biomedicines9050518)
Supplement: Supplementary file 1 [file biomedicines-09-00518-s001.zip › Table S1.pdf]

Supplementary Table S1. Food composition

	Experimental diet			
**Food sterol content**	**HFC**	**HFC + stanol**	**Chow**	**Chow + stanol**
Cholesterol (µg/mg)	1.75	1.62	0.017	0.025
Sitostanol (µg/mg)	0.006	11.7	0.249	21.7
Sitosterol (µg/mg)	0.007	0.145	0.211	0.534
Campestanol (µg/mg)	0.001	1.07	0.118	2.49
Campesterol (µg/mg)	0.003	0.086	0.062	0.086
Desmosterol (ng/dl)	23.7	21.7	0.714	0.383
Lathosterol (ng/mg)	10.3	7.63	0.219	0.378
27-OH cholesterol (ng/dl)	0.144	0.168	0.023	Below detection limit
7a-OH cholesterol (ng/dl)	13.5	22.2	0.554	0.132
