# Supplementary material for: Anti-Inflammatory Effects of Dietary Plant Stanol Supplementation Are Largely Dependent on the Intake of Cholesterol in a Mouse Model of Metabolic Inflammation
Source: Biomedicines. 2021 May 6;9(5):518. doi: 10.3390/biomedicines9050518 (PMC8148209; doi:10.3390/biomedicines9050518)
Supplement: Supplementary file 1 [file biomedicines-09-00518-s001.zip › Table S2.pdf]

Supplementary Table S2: bone marrow transplant efficiency

	**% *Ldlr*$^{-/-}$ DNA (mean)**	**SEM**
***Npc1*^{wt}-tp**	94.67	4.43
***Npc1*^{nih}-tp**	93.61	2.80
**All mice**	93.99	3.47
